# The role of the PD-1/PD-L1 immune checkpoint pathway in myocardial infarction: a review from pathophysiological mechanisms to therapeutic strategies

**DOI:** 10.3389/fcvm.2025.1691863

**Published:** 2025-11-26

**Authors:** Zilong Luo, Han Zhang, Junjie Zong, Jun Hu, Pinyan Huang, Yuqing Niu, Cheng Zhou, Song Wang, Dan Zhang

**Affiliations:** 1 Department of Cardiovascular Surgery, Union Hospital, Tongji Medical College, Huazhong University of Science and Technology, Wuhan, Hubei, China; 2 Center for Translational Medicine, Union Hospital, Tongji Medical College, Huazhong University of Science and Technology, Wuhan, Hubei, China; 3 NHC Key Laboratory of Organ Transplantation, Ministry of Education, Chinese Academy of Medical Sciences, Wuhan, Hubei, China; 4 Cancer Center, Union Hospital, Tongji Medical College, Huazhong University of Science and Technology, Wuhan Hubei, China; 5 Institute of Radiation Oncology, Union Hospital, Tongji Medical College, Huazhong University of Science and Technology, Wuhan Hubei, China

**Keywords:** PD-1, PD-L1, myocardial infarction, cardiac immune modulation, immune checkpointinhibitors, myocardial repair

## Abstract

Programmed cell death protein 1 (PD-1) and its ligand, programmed death-ligand 1 (PD-L1), are vital molecules in immune checkpoints, significantly impacting cancer treatment. Recent studies have increasingly highlighted the complex roles of the PD-1/PD-L1 pathway in cardiovascular diseases, particularly in myocardial infarction (MI). In addition to being involved in immune modulation and the inflammatory response post-myocardial ischemia, this pathway is also crucial for myocardial repair and regeneration. Additionally, the clinical application of PD-1/PD-L1 immune checkpoint inhibitors has led to groundbreaking advances in cancer treatment; however, concerns regarding cardiotoxicity and myocardial injury as adverse events have also been raised. This review systematically examines the pathophysiological mechanisms of the PD-1/PD-L1 pathway in myocardial infarction, explores its potential as a therapeutic target, and assesses the adverse cardiovascular reactions associated with existing immune checkpoint inhibitors and management strategies. The aim of this study was to provide a theoretical basis and clinical guidance for future immunotherapeutic approaches for treating myocardial infarction.

## 1 Introduction

Programmed cell death protein 1 (PD-1) and its ligand, programmed death-ligand 1 (PD-L1), have become central to modern cancer immunotherapy due to their regulatory effects on immune activity. PD-1, a receptor located on T cells, binds to PD-L1, which is often overexpressed on tumor cells, resulting in the suppression of T-cell function and enabling tumor immune escape. This interaction is a crucial pathway exploited by cancers to evade immune surveillance, making the PD-1/PD-L1 axis a prominent therapeutic target across multiple malignancies. Clinical investigations have demonstrated that blocking this pathway significantly improves overall survival and progression-free survival in patients with advanced cancers (1). Nonetheless, such therapies are associated with immune-related adverse events (irAEs), some of which involve the cardiovascular system (2). Therefore, clarifying the biological roles of the PD-1/PD-L1 pathway is vital, not only for understanding its relevance in oncology but also for exploring its implications in other diseases, including myocardial infarction (MI).

Myocardial infarction (MI), defined as the obstruction of coronary blood flow, results in ischemic damage followed by an inflammatory cascade essential for tissue repair and structural remodeling. The immune response in MI exhibits a paradoxical nature: while controlled inflammation promotes recovery, excessive or dysregulated activity can drive maladaptive remodeling and the progression to heart failure (3). Increasing attention has been directed toward immune checkpoints, including PD-1 and PD-L1, in this setting. Evidence indicates that the PD-1/PD-L1 axis may regulate post-MI immune activity, influencing the delicate equilibrium between beneficial repair processes and harmful inflammation (4). Elucidating this relationship could provide new therapeutic opportunities to optimize cardiac repair while limiting pathological immune responses.

With the rapid adoption of immune checkpoint inhibitors in oncology, their cardiovascular effects have become increasingly apparent. Although these agents have transformed cancer therapy, they are also linked to cardiovascular toxicities such as myocarditis, impaired cardiac function, and accelerated atherosclerotic disease (5). The precise mechanisms remain incompletely defined, but may involve aberrant T-cell activation following PD-1/PD-L1 blockade, resulting in heightened inflammatory signaling that destabilizes cardiovascular homeostasis (6). A deeper understanding of these mechanisms is essential to reduce cardiac risks and improve outcomes for patients receiving immunotherapy.

This review aims to systematically summarize the current understanding of the PD-1/PD-L1 pathway in the context of myocardial infarction, focusing on its potential regulatory roles and therapeutic implications. By evaluating the literature, we will assess the dual nature of immune responses during MI and the impact of PD-1/PD-L1 inhibition on cardiac outcomes. Furthermore, we explore the therapeutic potential of targeting this pathway in the treatment of MI, alongside the associated risks of cardiotoxicity that may arise from immunotherapy. Through this comprehensive analysis, we hope to provide insights that will inform clinical practice and guide future research directions at the intersection of immunology and cardiology.

## 2 Molecular mechanism and immune regulatory function of the PD-1/PD-L1 pathway

### 2.1 Structural and expression characteristics of PD-1 and PD-L1

The programmed cell death protein 1 (PD-1) receptor and its ligand, programmed death-ligand 1 (PD-L1), are central elements of the immune checkpoint pathway that regulate immune homeostasis. PD-1, a type I transmembrane receptor of the CD28 family, is mainly expressed on activated T cells, B cells, and natural killer (NK) cells. Structurally, it contains an extracellular region, a single transmembrane helix, and a cytoplasmic tail with two immunoreceptor tyrosine-based inhibitory motifs (ITIMs) and an immunoreceptor tyrosine-based switch motif (ITSM), which mediate its suppressive signaling (7). Engagement of PD-1 with its ligands, PD-L1 or PD-L2, attenuates T-cell receptor (TCR) signaling, thereby limiting T-cell activation and clonal expansion. PD-L1 itself is found on a wide range of cells, including tumor cells, macrophages, and dendritic cells, and its expression is strongly inducible by pro-inflammatory cytokines such as interferon-γ(IFN-γ) (8).

Importantly, PD-1 and PD-L1 are not expressed uniformly across tissues, a feature that becomes particularly relevant in myocardial infarction (MI) and other cardiovascular disorders. Within the heart, PD-L1 has been detected on cardiomyocytes as well as immune cells such as macrophages and T lymphocytes, implicating the PD-1/PD-L1 axis in the regulation of local inflammation and repair after ischemic damage (9). Stressors like hypoxia and inflammation can enhance PD-L1 expression in cardiac tissue, influencing immune activity during myocardial injury (10). Similarly, PD-1 expression has been observed in infiltrating leukocytes within the infarcted myocardium, suggesting it may contribute to the modulation of immune responses or even immune evasion in cardiac pathology (11).

In summary, the structural characteristics of PD-1 and PD-L1, along with their differential expression patterns in various tissues, particularly in the heart, highlight their significant roles in regulating immune responses. This regulation is crucial in the context of myocardial infarction, where the balance between immune activation and inhibition can influence outcomes related to cardiac repair and inflammation. Understanding these dynamics is essential for developing targeted therapeutic strategies that modulate the PD-1/PD-L1 pathway in cardiovascular diseases.

### 2.2 Immune suppression mechanisms and T-cell activity regulation

The PD-1/PD-L1 immune checkpoint axis is a key regulator of immune tolerance, particularly in myocardial infarction (MI). This pathway involves the interaction of programmed cell death protein 1 (PD-1) on T cells with its ligand, programmed death-ligand 1 (PD-L1), which is frequently elevated under pathological conditions such as ischemic heart disease. Binding of PD-L1 to PD-1 delivers inhibitory signals that induce T-cell dysfunction and exhaustion, thereby restraining immune activity. While this suppressive effect protects cardiac tissue from excessive inflammatory injury, it may also compromise immune surveillance against pathogens and malignancies. Notably, inflammatory mediators in the ischemic myocardium, including interferon-γ (IFN-γ), can further upregulate PD-L1 on cardiomyocytes and immune cells, enhancing local immunosuppression and potentially contributing to maladaptive remodeling and impaired repair after MI (12, 13).

Beyond promoting T-cell exhaustion, PD-1/PD-L1 signaling broadly limits T-cell activation, proliferation, and cytotoxicity. Inhibition of this pathway has been shown to reinvigorate T-cell function, restoring cytokine secretion and cytolytic activity of CD8+ T cells—responses critical for tumor clearance and pathogen defense. Clinical and experimental evidence indicates that PD-1 blockade can reestablish effective immune responses in chronic infections and cancers, underscoring its therapeutic potential (14, 15). Translating this concept to myocardial infarction, interrupting PD-1/PD-L1 signaling could, in theory, augment T-cell–mediated repair and improve post-ischemic outcomes. However, excessive immune reactivation risks uncontrolled inflammation, which may aggravate tissue injury, highlighting the need for careful modulation of this pathway.

The regulation of T-cell responses in the infarcted heart is further shaped by other immune checkpoints and suppressive cell populations. For instance, PD-1 acts in concert with inhibitory receptors such as CTLA-4, collectively influencing post-MI immune dynamics. Moreover, regulatory T cells (Tregs) and myeloid-derived suppressor cells (MDSCs) contribute to an immunosuppressive milieu that further dampens effector T-cell activity. Dissecting these complex cellular and molecular interactions is crucial for designing immunotherapeutic approaches that can selectively enhance cardiac repair while limiting collateral damage. Future investigations should aim to delineate the contributions of distinct immune subsets and checkpoint pathways during cardiac ischemia, thereby informing novel strategies that harness immune modulation for myocardial protection and regeneration (16, 17).

### 2.3 The role of PD-1/PD-L1 in the myocardial immune microenvironment

The PD-1/PD-L1 signaling pathway plays a crucial role in regulating immune responses within the myocardial microenvironment, particularly following ischemic events such as myocardial infarction. The dynamic expression of PD-1 and its ligand PD-L1 is significantly influenced by the infiltration of various immune cells into the myocardium during ischemia. Studies have shown that after myocardial ischemia, there is an acute influx of immune cells, including T cells, macrophages, and dendritic cells, which contribute to the inflammatory response and subsequent tissue remodeling (18). The upregulation of PD-1 on T cells and PD-L1 on cardiomyocytes and antigen-presenting cells serves as a critical mechanism to limit excessive inflammation and prevent autoimmunity, thereby promoting a state of immune tolerance. This balance is essential, as excessive inflammation can lead to further myocardial damage and heart failure, whereas an insufficient immune response may allow for persistent viral infections or tumor progression. The intricate interplay between immune cell infiltration and PD-1/PD-L1 expression highlights the dual role of this pathway in both protective and pathogenic processes within the heart (19).

Moreover, the regulation of immune tolerance vs. inflammatory responses in the myocardium is a complex process that involves not only the PD-1/PD-L1 axis but also other immune checkpoints and signaling pathways. For example, the engagement of PD-1 on T cells inhibits their activation and proliferation, which can be beneficial in preventing tissue damage during acute inflammation. However, in the context of chronic inflammation or persistent antigen exposure, this pathway may contribute to T-cell exhaustion, leading to impaired immune responses against pathogens or tumors (20). The balance between immune tolerance and inflammation is further complicated by the presence of other immune modulators and the local cytokine milieu, which can either enhance or inhibit PD-1/PD-L1 interactions. Understanding these dynamics is crucial for developing therapeutic strategies aimed at modulating the immune response in myocardial ischemia and other cardiovascular diseases (21).

Recent research has explored the potential of targeting the PD-1/PD-L1 pathway as a therapeutic strategy for MI. By inhibiting this pathway, it may be possible to enhance T-cell responses and promote tissue repair following ischemic injury. However, this approach must be carefully balanced against the risk of exacerbating inflammation and causing further myocardial damage. The timing of intervention, the specific immune context, and the presence of other costimulatory or inhibitory signals are all critical factors that must be considered when developing therapies targeting the PD-1/PD-L1 axis in the myocardial immune microenvironment (22). As our understanding of the immunological landscape of the heart continues to evolve, it becomes increasingly clear that the PD-1/PD-L1 pathway is a key player in modulating both protective and pathological immune responses, with significant implications for the management of ischemic heart disease and other related conditions.

## 3 Expression characteristics of PD-L1 in the myocardial tissue of patients with myocardial infarction

### 3.1 Changes in PD-L1 expression after myocardial ischemia

The expression of programmed death-ligand 1 (PD-L1) in myocardial tissue after ischemic injury has become a focus of investigation because of its role in immune regulation and potential therapeutic relevance. Both immunohistochemical and molecular studies reveal substantial alterations in PD-L1 levels during myocardial ischemia. In particular, patients with acute myocardial infarction (AMI) exhibit significantly higher myocardial PD-L1 expression compared with healthy individuals. This increase correlates with the presence of regulatory T cells (Tregs) and myeloid-derived suppressor cells (MDSCs), both of which express PD-L1 and contribute to shaping immune responses after ischemic injury (23). The recruitment of these immune cell populations and their associated PD-L1 upregulation appear to provide a protective mechanism by attenuating immune-mediated damage while facilitating tissue repair. Furthermore, PD-L1 expression differs across cardiovascular conditions: it is often elevated in ischemic heart disease, especially in necrotic regions, but markedly reduced in dilated cardiomyopathy, indicating that distinct disease mechanisms may dictate PD-L1 dynamics (24). These patterns suggest that PD-L1 could serve as both a biomarker of disease state and a regulator of cardiac immune responses.

The functional significance of PD-L1 in cardiac disorders has also been linked to clinical outcomes. Evidence demonstrates an inverse association between PD-L1 expression and left ventricular ejection fraction (LVEF) in patients with ischemic heart disease (24). This finding implies that elevated PD-L1 may accompany impaired cardiac performance, possibly reflecting chronic inflammatory activity characteristic of ischemic disease. At the same time, PD-L1 upregulation under ischemic stress may act as a compensatory pathway to dampen excessive immune activation, thereby limiting additional myocardial injury. The dynamic regulation of PD-L1, in concert with infiltrating immune cells, highlights a complex immunological environment that influences both immediate ischemic responses and long-term cardiac remodeling.

Overall, alterations in PD-L1 expression following myocardial ischemia reflect the close interconnection between immune activity and cardiac pathology. The observed upregulation in AMI supports a context-dependent protective role, though its precise impact on long-term function requires clarification. Dissecting how PD-L1 expression varies across different cardiovascular diseases may inform strategies aimed at modulating immunity for improved cardiac repair. Future studies should clarify the mechanisms underlying PD-L1 regulation and evaluate its implications for therapeutic interventions, particularly in the setting of immune checkpoint blockade, where cardiovascular toxicity remains a concern (6).

### 3.2 Correlation between PD-L1 expression and cardiac function indicators

The role of PD-L1 in regulating cardiac function has gained increasing attention, especially in myocardial infarction (MI). Experimental and clinical data suggest a significant association between PD-L1 expression and key cardiac parameters, including left ventricular ejection fraction (LVEF) and end-diastolic volume (EDV). For instance, an investigation in female BALB/c mice subjected to MI showed that administration of PD-L1 monoclonal antibodies (mAbs) improved LVEF and reduced myocardial damage, as confirmed by echocardiographic and histopathological evaluation (25). These beneficial effects were linked to modulation of the CD47/SHP2/SIRPα/SYK/FcγR pathway in tumor-associated macrophages, indicating that PD-L1 may contribute to post-ischemic cardiac repair. Increased PD-L1 expression in cardiac tissue has also been correlated with stronger anti-inflammatory activity, facilitating myocardial healing and improved functional outcomes. Clinically, higher PD-L1 levels in patients are often associated with better cardiac performance, suggesting its potential utility as a biomarker for recovery following ischemic injury (26).

Beyond these functional associations, the mechanisms underlying PD-L1-mediated cardioprotection remain under active investigation. PD-L1 suppresses T-cell activation, thereby attenuating inflammation and limiting immune-mediated myocardial injury (27). This immune-regulatory effect is particularly relevant after ischemic stress, where excessive inflammation can worsen tissue damage. Furthermore, PD-L1 expression influences macrophage polarization toward an M2 reparative phenotype, which promotes anti-inflammatory signaling and tissue regeneration (26).

In summary, the relationship between PD-L1 expression and cardiac function indicators such as LVEF and EDV reflects a complex interplay between direct myocardial protection and immune regulation. Clinically, PD-L1 emerges as both a promising biomarker of cardiac performance and a potential therapeutic target for improving outcomes after ischemic injury. Future studies should clarify the underlying molecular pathways and assess the translational potential of PD-L1–based interventions in cardiovascular disease.

## 4 The regulatory role of the PD-1/PD-L1 pathway in the immune inflammation of myocardial infarction

### 4.1 Recruitment and activation of immunosuppressive cell populations

The dynamic fluctuations of myeloid-derived suppressor cells (MDSCs) and regulatory T cells (Tregs) after myocardial infarction (MI) are central to shaping the post-injury immune milieu. MDSCs, encompassing both suppressor subsets and their precursors, undergo substantial expansion in response to pathological stressors such as ischemic heart disease. During MI, the accumulation of MDSCs fosters an immunosuppressive environment that can limit reparative immunity and contribute to maladaptive remodeling. Their increase is thought to be driven by damage-associated molecular patterns (DAMPs) and inflammatory cytokines released during ischemia. Importantly, these cells actively suppress T-cell activation and promote Treg differentiation, rather than simply expanding passively. In parallel, Tregs themselves are enriched in the infarcted heart, playing a dual role: while they curb excessive inflammation and reduce tissue injury, they may also restrain immune responses required for optimal regeneration. This bidirectional influence of MDSCs and Tregs underscores the importance of deciphering their recruitment and function for the development of therapies that support effective cardiac repair after MI (28).

The PD-1/PD-L1 axis is a key mediator of MDSC and Treg activity in the infarcted heart. PD-L1, expressed on both cell types, binds to PD-1 on T cells and suppresses their activation and proliferation. This mechanism is amplified following MI, where myocardial PD-L1 expression rises, providing a means of protecting tissue from immune-mediated injury. Enhanced PD-L1 expression on MDSCs boosts their suppressive functions, allowing more efficient inhibition of effector T-cell responses that would otherwise contribute to tissue clearance and repair. Similarly, Tregs depend on PD-L1 to sustain their immunosuppressive phenotype, reinforcing an environment supportive of repair but with the risk of prolonged or excessive immune suppression. This duality highlights a delicate balance: PD-L1–driven suppression shields the myocardium from excessive inflammation, yet it may also impede immune activation necessary for regeneration. Therapeutic modulation of the PD-1/PD-L1 pathway, therefore, holds promise to both enhance beneficial immune responses and reduce the likelihood of adverse remodeling after MI (29, 30).

### 4.2 Interaction of inflammatory factors and immune signaling pathways

The PD-1/PD-L1 immune checkpoint pathway is a central regulator of inflammation and apoptosis, particularly relevant in myocardial infarction (MI). Its interaction with inflammatory mediators, including nuclear factor kappa B (NF-κB) and caspase-3, plays an essential role in immune balance and apoptotic control within cardiac tissue. NF-κB, a key transcription factor, drives the production of proinflammatory cytokines and immune checkpoint molecules such as PD-L1 when activated. In MI, this mechanism is especially significant because controlled inflammation supports tissue repair, whereas unchecked responses may trigger pathological remodeling. Evidence indicates that inflammatory cytokines can induce PD-L1 expression, which in turn promotes cardiomyocyte survival by limiting caspase-3–dependent apoptosis under stress conditions (31). Additionally, engagement of PD-L1 with PD-1 on T cells suppresses immune activity and dampens inflammation, thereby reducing acute myocardial injury during ischemic stress (32). These observations suggest that PD-1/PD-L1 signaling may function as a protective mechanism by balancing cell survival with immune regulation in the infarcted heart.

Beyond acute protection, PD-1/PD-L1 signaling also influences apoptosis and fibrotic remodeling after ischemic injury. Activation of this pathway reduces T-cell–mediated cytotoxicity, which preserves cardiomyocyte integrity but can limit necessary immune surveillance. As a consequence, fibrotic remodeling may prevail, contributing to long-term cardiac dysfunction and heart failure (33). Moreover, cytokines such as IL-6 and TNF-α can further enhance PD-L1 expression, reinforcing a feedback loop that sustains immune suppression and may aggravate fibrosis (34). Thus, while the PD-1/PD-L1 axis mitigates early apoptosis and acute inflammation, it may also predispose the myocardium to maladaptive structural changes.

In conclusion, PD-1/PD-L1 signaling represents a dual-edged regulator of immune and apoptotic processes in MI. Its capacity to shield cardiomyocytes during acute ischemia is counterbalanced by its potential to foster fibrosis and adverse remodeling in the chronic phase. Future therapeutic strategies will likely require precise modulation of this pathway to maximize myocardial protection while minimizing long-term risks such as fibrosis and heart failure.

### 4.3 Mendelian randomization analysis reveals the association of PD-1/PD-L1 with CHD

The association between programmed cell death protein-1 (PD-1) and chronic ischemic heart disease (CHD) has been further clarified through Mendelian randomization (MR) analysis, a powerful approach for inferring causality in complex traits. Recent evidence suggests a bidirectional causal link between PD-1 expression and CHD risk, indicating that genetic variations influencing PD-1 levels may alter susceptibility to the disease. Specifically, MR analysis reported an odds ratio (OR) of 0.997 (95% CI, 0.995–0.999; *P* = 0.009), implying that elevated PD-1 expression is correlated with a reduced risk of CHD (35). These results highlight the potential of PD-1 as both a biomarker and a contributor to the pathophysiology of ischemic heart disease. Interestingly, reverse MR analysis showed that CHD itself may reduce PD-1 expression, with a beta estimate of −3.1 (95% CI, −6.017 to −0.183; *P* = 0.037), suggesting a feedback mechanism whereby the disease state influences immune checkpoint regulation. Collectively, these findings underscore PD-1's dual role as a protective factor and a target for future therapeutic strategies aimed at reducing cardiovascular risk.

In addition to PD-1, programmed death-ligand 1 (PD-L1) has also been implicated in the progression of chronic ischemic heart disease. MR analysis identified a significant inverse association, with a beta coefficient of −3.269 (95% CI, −6.197 to −0.341; *P* = 0.029) (35), supporting the notion that higher PD-L1 levels may exert cardioprotective effects. This protective influence is thought to stem from PD-L1's capacity to suppress T-cell activation, promote immune tolerance, and attenuate persistent inflammation within the cardiac microenvironment. Gene set enrichment analysis (GSEA) further demonstrated that PD-L1-related signaling and PD-1 checkpoint pathways were downregulated in CHD (36), implying potential impairment of immune checkpoint function that may exacerbate inflammatory injury. Taken together, these data emphasize a complex interplay between PD-1, PD-L1, and ischemic heart disease, and suggest that therapeutic modulation of these immune checkpoint pathways could represent a promising strategy for the prevention and treatment of cardiovascular disorders.

### 4.4 Gene set enrichment analysis (GSEA) for validation of relevant signaling pathways

The role of the PD-1/PD-L1 immune checkpoint pathway in coronary heart disease (CHD) has garnered significant attention, particularly in understanding its gene expression profiles. Recent findings indicate that the expression of immune checkpoint pathways, specifically PD-1 and PD-L1, is notably downregulated in patients with CHD. This downregulation was substantiated through gene set enrichment analysis (GSEA), which utilized gene expression profiles from the Gene Expression Omnibus (GEO) database, specifically the GSE71226 dataset. The analysis revealed a significant causal association between PD-1 expression and chronic ischemic heart disease, with an odds ratio of 0.997, indicating a protective role of PD-1 in the context of CHD (35). Furthermore, the analysis demonstrated that both PD-1 and PD-L1 exhibited negative beta coefficients in relation to chronic ischemic heart disease, suggesting that lower levels of these proteins are correlated with disease progression. These findings emphasize that the downregulation of these immune checkpoints may contribute to the pathophysiological mechanisms underlying CHD, potentially leading to increased myocardial injury and inflammation.

In addition to confirming the downregulation of the PD-1/PD-L1 pathway, GSEA also revealed several core genes associated with this pathway and their functional annotations. The KEGG pathway analysis highlighted the “PD-L1 expression and PD-1 checkpoint pathway in cancer,” which, despite its primary focus on oncological contexts, sheds light on the immunological alterations occurring in CHD. The core genes identified through this analysis play crucial roles in immune regulation and inflammatory responses, which are vital in the context of myocardial ischemia and reperfusion injury. The downregulation of these genes in CHD patients suggests potential impairment of the immune response, which could exacerbate the progression of coronary artery disease. This impairment may lead to a reduced ability to modulate inflammation and apoptosis, thus contributing to the adverse outcomes associated with myocardial infarction and other ischemic heart conditions (37).

Functional annotation of the core genes indicated their participation in multiple biological processes, such as T-cell activation, cytokine-mediated signaling, and the regulation of apoptosis. Genes that are normally induced by PD-1 signaling appear to be critical for maintaining immune balance and limiting excessive inflammation during myocardial ischemia. Conversely, reduced expression of these genes may reflect impaired immune regulation, potentially aggravating the pathogenesis of chronic ischemic heart disease (CHD). These findings emphasize that the PD-1/PD-L1 pathway is not only a therapeutic candidate but also a fundamental element for interpreting the immune-related mechanisms underlying CHD (38).

In summary, gene set enrichment analysis (GSEA) provides strong evidence that the PD-1/PD-L1 checkpoint pathway is downregulated in CHD, along with the identification of associated core genes and their biological functions. These results deepen the understanding of immune dysregulation in CHD and support the development of innovative therapeutic approaches that target this pathway to optimize clinical outcomes. Future research focusing on manipulating PD-1/PD-L1 signaling in patients with significant myocardial ischemia may offer new opportunities for improving disease management (39).

## 5 Cardiotoxicity and myocardial infarction risk associated with immune checkpoint inhibitors (ICIs)

### 5.1 Clinical trials and cohort studies on cardiovascular adverse event statistics

The introduction of ICIs, especially those targeting PD-1 and PD-L1, has transformed cancer treatment but has also brought attention to their potential cardiovascular toxicities. Reports from clinical studies and observational cohorts have identified a spectrum of cardiovascular adverse events (CVAEs), including myocarditis, heart failure, arrhythmias, and acute myocardial infarction, with varying frequencies (Table 1). A systematic review and meta-analysis estimated the incidence of myocarditis in ICI-treated patients to be about 0.5% (40), while arrhythmias were observed more frequently, occurring in roughly 4.6% of cases (40). In addition, evidence from a cohort study showed that 10.3% of patients developed major adverse cardiovascular events (MACEs) during a median follow-up of 13 months, underscoring the importance of close cardiovascular surveillance in individuals receiving ICIs (41). Chemotherapy increases the risk of cardiovascular events (myocarditis, MI, arrhythmias) when combined with ICIs, possibly via PD-1/PD-L1 pathway modulation.

**Table 1:** Adverse effects of ICIs.

ICI Type	Combination strategy	Reported cardiovascular adverse events
PD-1 inhibitors (e.g., nivolumab)	Monotherapy	Myocarditis (∼0.5%), arrhythmias (∼4.6%), major adverse cardiovascular events (MACEs) in 10.3% during 13-month follow-up
PD-L1 inhibitors (e.g., atezolizumab)	Monotherapy	Increased risk of myocardial infarction and heart failure compared to PD-1 inhibitors; higher rate of MACE in pharmacovigilance data
PD-1 + CTLA-4 inhibitors (e.g., nivolumab + ipilimumab)	Combination immunotherapy	Higher risk of grade 5 arrhythmias (OR ≈ 3.90), increased myocarditis incidence (0.27% vs. 0.06% for PD-1 monotherapy)

The incidence of specific CVAEs appears to differ between PD-1 inhibitors and PD-L1 inhibitors. For example, a meta-analysis demonstrated that PD-1 inhibitors combined with CTLA-4 inhibitors significantly increased the risk of grade 5 arrhythmias compared with monotherapy with PD-1 inhibitors alone (42). In contrast, PD-1 inhibitors are associated with a greater incidence of myocardial infarction and heart failure than PD-L1 inhibitors are, indicating a potential difference in cardiac safety profiles between these classes of ICIs (43). Additionally, a study focusing on real-world data from patients treated with ICIs reported that the overall incidence of CVAEs was approximately 8%, with arrhythmias being the most common adverse event (44).

The differential cardiovascular safety profiles of PD-1 and PD-L1 inhibitors may be attributed to their distinct mechanisms of action and the immune responses they elicit. PD-1 inhibitors primarily enhance T-cell activity against tumors, which may inadvertently lead to increased inflammation and subsequent cardiovascular risk. On the other hand, PD-L1 inhibitors may have a more pronounced effect on the vascular endothelium, contributing to a higher incidence of ischemic events (45). This distinction underscores the importance of individualized treatment strategies and the necessity for ongoing cardiovascular assessment in patients receiving ICI therapy.

In summary, clinical trials and cohort studies highlight the incidence of cardiovascular adverse events associated with PD-1 and PD-L1 inhibitors, with notable differences in safety profiles. These data suggest that while both classes of ICIs carry risks for serious cardiovascular complications, the nature and frequency of these events may vary significantly. As the use of ICIs continues to expand, further research is essential to elucidate the underlying mechanisms of these adverse events and to develop effective monitoring and management strategies for at-risk patients.

### 5.2 Clinical manifestations and diagnostic challenges of immune-related myocarditis

ICI-related myocarditis is a rare but potentially fatal complication of cancer immunotherapy that poses significant diagnostic challenges owing to its nonspecific clinical presentation. Patients with ICI-related myocarditis often exhibit symptoms such as fatigue, chest pain, palpitations, and dyspnea, which can be easily mistaken for other conditions, including acute coronary syndrome (ACS) or exacerbations of preexisting cardiac diseases (46). The clinical symptoms may vary widely among patients, with some presenting with severe manifestations such as cardiogenic shock or arrhythmias, whereas others may show only mild symptoms or even remain asymptomatic despite significant myocardial injury (47). The diagnosis is further complicated by the potential overlap with other immune-related adverse events (irAEs), such as myositis or myasthenia gravis, which can cooccur, leading to a more complex clinical picture (48). Electrocardiographic (ECG) changes, such as new-onset arrhythmias, ST-segment changes, or conduction abnormalities, are often observed in patients with ICI-related myocarditis, but these findings are not specific (49). Additionally, echocardiography may reveal a reduced left ventricular ejection fraction (LVEF) or wall motion abnormalities, but it may not always correlate with the severity of symptoms or troponin elevation (46, 50).

The diagnostic process for ICI-related myocarditis typically relies on a combination of clinical evaluation, biomarker assessment, and imaging studies. Cardiac biomarkers, particularly troponins, are critical in the diagnosis of myocarditis, as elevated levels indicate myocardial injury (47). However, the timing of biomarker elevation can vary, and in some cases, patients may present with elevated troponin levels without clear clinical symptoms, complicating the diagnosis (51). Imaging modalities such as cardiac magnetic resonance imaging (CMR) have emerged as valuable tools for diagnosing ICI-related myocarditis, offering insights into myocardial inflammation and edema that may not be apparent on echocardiography (52). CMR can detect characteristic patterns of late gadolinium enhancement, which are indicative of myocarditis, and can help differentiate it from other cardiac conditions (53). Despite these advancements, the diagnosis remains challenging, particularly in distinguishing ICI-related myocarditis from acute ST-segment elevation myocardial infarction (STEMI), as both conditions can present with similar clinical features, including chest pain and elevated cardiac enzymes (50).

Case analyses have revealed instances where patients with ICI-related myocarditis were initially misdiagnosed with STEMI, leading to delays in appropriate treatment (46, 48). For example, in a reported case, a patient receiving ICI therapy developed symptoms consistent with acute myocardial infarction and was only later diagnosed with myocarditis after further evaluation revealed significant troponin elevation and abnormal imaging findings (49). This underscores the importance of maintaining a high index of suspicion for myocarditis in patients receiving ICI therapy, especially when faced with atypical presentations. The overlap of symptoms and the potential for rapid clinical deterioration necessitate a multidisciplinary approach involving oncologists, cardiologists, and other specialists to ensure timely recognition and management of this serious adverse event (47). In summary, the clinical manifestations and diagnostic challenges of ICI-related myocarditis require careful consideration of the unique presentation of each patient, along with the use of advanced diagnostic tools to differentiate it from other cardiac conditions effectively.

### 5.3 Risk of thromboembolic events associated with combination therapy with immune checkpoint inhibitors

The use of ICIs, particularly those targeting the PD-1/PD-L1 pathway, has significantly transformed cancer treatment paradigms. However, the combination of ICIs with other therapeutic agents, such as CTLA-4 inhibitors, raises concerns regarding the risk of thromboembolic events (TEEs). Recent studies have shown that patients receiving ICI therapy, especially combination regimens, exhibit increased incidences of both venous thromboembolism (VTE) and arterial thromboembolism (ATE). For example, a meta-analysis indicated that the cumulative incidence of VTE in patients receiving ICIs can reach 20%, with combination therapies resulting in even higher rates than monotherapies (54). This elevated risk is particularly pronounced in certain cancer types, such as melanoma and lung cancer, where the combination of PD-1 inhibitors with CTLA-4 inhibitors has been associated with a notable increase in TEEs (55). The underlying mechanisms contributing to this prothrombotic state may involve immune dysregulation and increased inflammatory responses triggered by the activation of immune pathways, which can lead to endothelial dysfunction and a hypercoagulable state (56). Moreover, factors such as preexisting conditions, including a history of thromboembolic events, can further exacerbate the risk in these patients, necessitating careful monitoring and risk assessment during treatment (57).

The modulation of thrombotic risk by tumor type is an essential consideration in the management of patients receiving ICIs. Different cancers present varying baseline risks for thrombosis, which can be influenced by the tumor microenvironment and the specific immunological responses elicited by the cancer. For example, patients with lung cancer have been shown to have a greater incidence of VTE than those with other malignancies do, particularly when treated with ICIs (58, 59). The Khorana risk score, which is traditionally used to assess VTE risk in cancer patients, has been evaluated for its ability to predict thrombotic events in patients treated with ICIs. Studies suggest that while the Khorana score can stratify risk effectively in chemotherapy-treated patients, its utility in the context of ICIs may be limited, indicating a need for tailored risk assessment models that consider the unique pathophysiological changes induced by immunotherapy (60). Because the Khorana score was derived from cohorts with limited representation of malignant hemopathies and may not be calibrated for patients such as those with Hodgkin lymphoma or multiple myeloma receiving ICIs, future studies should evaluate global hemostatic assays—particularly thrombin generation testing—in combination with clinical risk factors as a personalized approach to thrombotic risk stratification in this population. Furthermore, the interplay between tumor characteristics, such as histological subtype and stage, and the risk of thrombosis underscores the importance of individualized treatment approaches. For example, patients with adeno carcinoma have been observed to have a distinct risk profile for thromboembolic events, which may necessitate different management strategies than those for other cancer types (61).

In conclusion, the combination of PD-1/PD-L1 inhibitors with CTLA-4 inhibitors presents a complex landscape regarding thromboembolic risk, influenced by both the therapeutic regimen and the underlying tumor type. As the use of ICIs continues to expand, understanding the nuances of thrombotic risks associated with these therapies will be crucial for optimizing patient outcomes and minimizing adverse events. Future research should focus on elucidating the mechanisms behind ICI-associated thrombosis and developing effective prophylactic strategies to mitigate this risk in vulnerable patient populations.

### 5.4 Synergistic effects of PD-L1/Akt in stem cell therapy

The combined overexpression of programmed death-ligand 1 (PD-L1) and Akt in adipose-derived mesenchymal stem cells (AdMSCs) has emerged as a promising strategy for myocardial infarction (MI) therapy. Recent findings suggest that genetic modification of AdMSCs to co-express PD-L1 and Akt enhances their reparative potential by improving cardiac function and increasing resistance to oxidative stress during post-MI recovery (62). This dual modification strengthens both cell survival and immunomodulatory capacity. PD-L1 is central to immune regulation, helping to mitigate the inflammatory milieu that aggravates myocardial injury, while activation of the Akt pathway promotes survival and proliferation of stem cells, both of which are essential for regeneration. Preclinical evidence indicates that PD-L1/Akt-overexpressing AdMSCs display improved tolerance to reactive oxygen species (ROS), a key challenge within the ischemic cardiac microenvironment (63).

Animal studies have further validated these observations, showing that transplantation of modified AdMSCs improves myocardial performance, demonstrated by higher left ventricular ejection fraction and reduced infarct size. The mechanisms appear to involve two major processes: activation of the PI3K/Akt pathway to suppress cardiomyocyte apoptosis, and PD-L1-mediated modulation of immune responses that fosters regulatory T-cell differentiation. Together, these processes not only preserve cardiomyocyte viability but also attenuate inflammatory damage, thereby promoting structural and functional recovery of the heart (64).

Additional experimental evidence reinforces these findings. Modified AdMSCs overexpressing PD-L1 and Akt significantly reduce ROS accumulation and better maintain myocardial architecture, as confirmed by histological analysis and functional testing (65). These results highlight the synergistic contribution of PD-L1 and Akt pathways to improving the therapeutic efficacy of stem cell–based interventions for MI.

In summary, engineering AdMSCs to co-express PD-L1 and Akt represents an innovative stem cell therapy approach for ischemic heart disease. This strategy improves stem cell viability and cardiac reparative effects while simultaneously reshaping the immune environment to favor tissue repair. Future investigations should aim to refine delivery techniques for these engineered cells and further dissect the molecular mechanisms underlying their cardioprotective actions, ultimately advancing regenerative treatments for patients with MI.

### 5.5 Immune regulation and mechanisms promoting myocardial repair

The induction of CD25+ regulatory T cells (Tregs), such as those observed in myocardial infarction (MI), plays a pivotal role in myocardial protection following ischemic injury. Tregs are known for their immunosuppressive properties, which are crucial in moderating the inflammatory response that can exacerbate cardiac damage post-MI. These cells facilitate the resolution of inflammation by secreting anti-inflammatory cytokines, such as IL-10 and TGF-β, which not only inhibit proinflammatory pathways but also promote tissue repair mechanisms (66). Studies have shown that the presence of Tregs in the ischemic heart can lead to reduced cardiomyocyte apoptosis and increased survival of cardiac cells, thereby preserving cardiac function. Moreover, Tregs can modulate the activity of other immune cells, including macrophages and dendritic cells, steering them toward a reparative phenotype rather than a destructive phenotype. This shift is particularly important, as macrophages can adopt either proinflammatory (M1) or anti-inflammatory (M2) states depending on the local cytokine milieu. The balance between these states is critical for effective myocardial repair, as M2 macrophages are associated with tissue regeneration and fibrosis resolution. Therefore, enhancing the induction of CD25+ Tregs in the context of MI could represent a therapeutic strategy to harness their protective effects, potentially leading to improved outcomes in patients suffering from cardiac ischemia (67).

**Figure 1:**
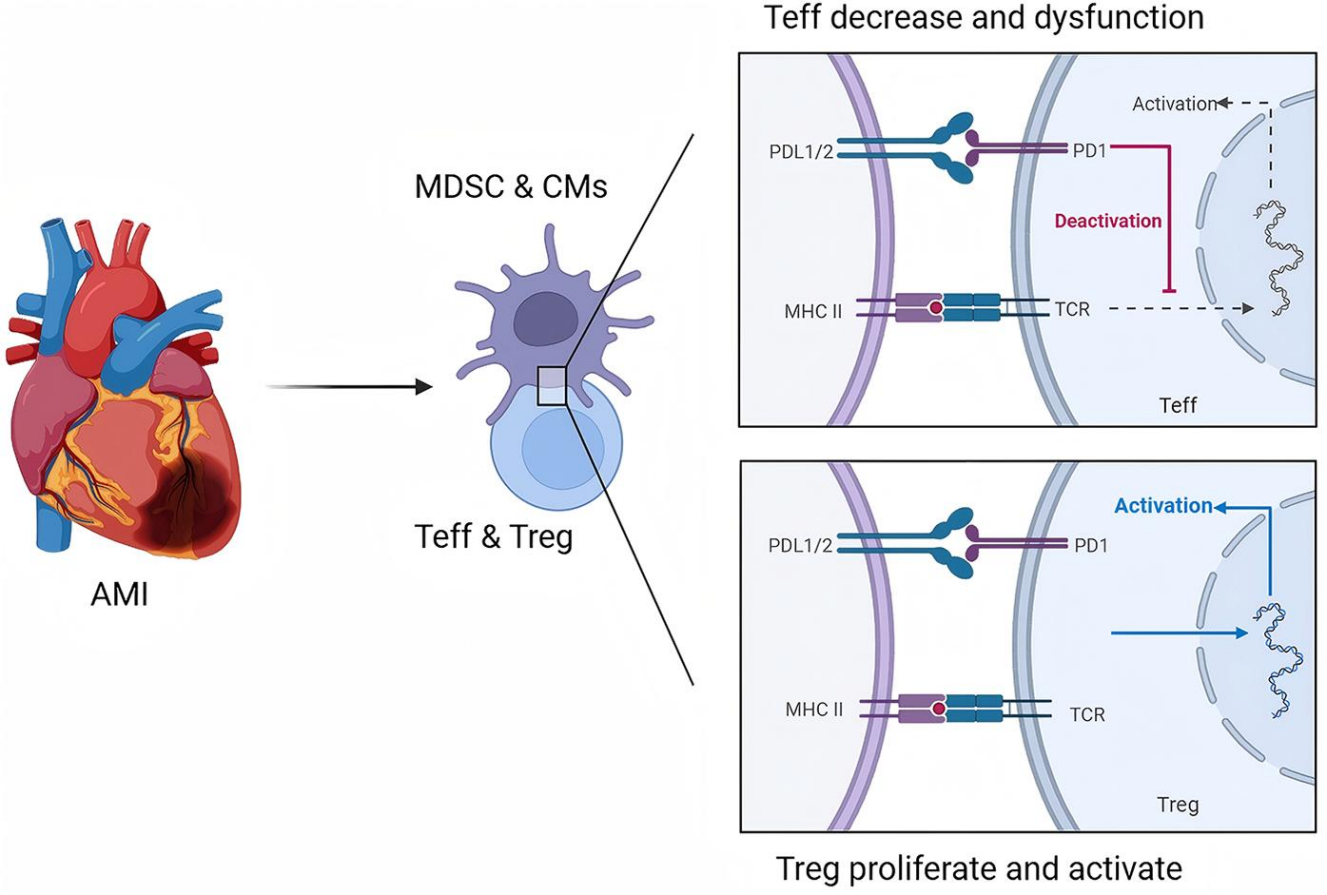
Role of the PD-1/PD-L1 pathway in regulating T-cell immunity during AMI. This figure is created with BioRender.com.

In addition to the role of Tregs, the molecular mechanisms underlying the suppression of inflammatory responses and the reduction in apoptosis in the heart are complex and multifaceted. One key aspect involves the modulation of immune checkpoint pathways, particularly the PD-1/PD-L1 axis, which serves to inhibit excessive T-cell activation and proliferation during inflammatory responses. In the context of MI, the upregulation of PD-L1 on cardiac cells can lead to the inhibition of cytotoxic T-cell activity, thereby reducing myocardial injury (30). This immune checkpoint pathway not only limits inflammation but also promotes a more favorable environment for cardiac repair. Furthermore, the inhibition of proapoptotic signaling pathways through the activation of antiapoptotic factors, such as Bcl-2, has been implicated in the survival of cardiomyocytes following ischemic injury. The interplay between immune regulation and apoptotic pathways is critical, as excessive apoptosis can lead to adverse remodeling of the heart and subsequent heart failure (68, 69). Thus, targeting these molecular mechanisms, such as through pharmacological agents that enhance Treg function or inhibit the PD-1/PD-L1 pathway, may provide new avenues for therapeutic intervention in patients with MI. Overall, understanding the intricate balance between immune regulation and myocardial repair processes is essential for developing effective strategies to mitigate cardiac damage and promote recovery following ischemic events (Figure 1).

## 6 Regulation of atherosclerosis and myocardial infarction risk via the immune checkpoint pathway

### 6.1 Evidence from animal experiments on immune checkpoint inhibitors promoting atherosclerosis

The role of ICIs in promoting atherosclerosis has been substantiated by various animal studies, particularly those utilizing atherosclerotic mouse models such as ApoE−/− and Ldlr−/− mice. These studies demonstrated that the inhibition of immune checkpoints, specifically PD-1 and CTLA-4, leads to significant alterations in atherosclerotic plaque characteristics. For example, a systematic review and meta-analysis revealed that the inhibition of PD-1 resulted in a 53% increase in atherosclerotic plaque size in treated mice compared with control mice, indicating a robust correlation between immune checkpoint inhibition and accelerated atherosclerosis (30). The composition of these plaques was notably different, exhibiting a greater abundance of immune cells, particularly CD4+ T cells, CD8+ T cells, and macrophages. This infiltration suggests a shift toward a more inflammatory state within the plaques, which is detrimental to cardiovascular health. Furthermore, when immune checkpoint proteins were stimulated, a contrasting effect was observed, with a reduction in plaque size of 28% in treated mice, accompanied by a decrease in the presence of macrophages and T cells (30). Moreover, although direct evidence is limited, initial studies indicate that concurrent infections or inflammatory processes in other organs (e.g., pneumonitis during PD-1/PD-L1 inhibitor therapy) can amplify systemic immune activation and potentially exacerbate the cardiotoxic effects of PD-1/PD-L1 immune checkpoint inhibitors. These findings underscore the dual role of immune checkpoints in regulating inflammation and atherosclerosis, where their inhibition exacerbates plaque formation and inflammation, whereas their activation appears to confer protective effects (70).

In addition to plaque size, the inflammatory status of atherosclerotic lesions has been a focal point of investigation. Studies have utilized advanced imaging techniques, such as positron emission tomography (PET), to visualize the dynamics of immune cell infiltration in response to ICIs. For example, the use of a CCR2-targeted radiotracer resulted in significantly elevated levels of CCR2+ proinflammatory macrophages in the atherosclerotic arteries of mice treated with anti-PD-1 antibodies than in those of control mice (71). This increase in macrophage infiltration is correlated with heightened inflammatory responses, as evidenced by elevated levels of proinflammatory cytokines and tissue damage markers. The underlying mechanism appears to involve the activation of IFNγ signaling pathways, which have been implicated in driving inflammation and promoting atherosclerotic plaque progression following ICI treatment (71). These findings indicate that immune checkpoint inhibition not only influences plaque size but also significantly alters the inflammatory milieu within atherosclerotic lesions, potentially leading to adverse cardiovascular events such as myocardial infarction and ischemic stroke.

The implications of these animal studies extend to clinical settings, where an increased risk of cardiovascular events in patients receiving ICI therapy has been reported. Accelerated atherosclerosis linked to immune checkpoint inhibition raises critical questions regarding the management of cancer patients, particularly those with preexisting cardiovascular conditions (72). This highlights the necessity for vigilant cardiovascular monitoring in this patient population, as the inflammatory changes induced by ICIs can have profound implications for heart health. Moreover, these studies suggest potential therapeutic avenues, such as the use of anti-inflammatory agents or strategies aimed at modulating immune responses, to mitigate the cardiovascular risks associated with ICIs. Overall, the evidence from animal experiments underscores the complex interplay between immune modulation and cardiovascular health, emphasizing the need for further research to elucidate the mechanisms at play and to develop effective strategies to protect against ICI-induced atherosclerosis.

### 6.2 Clinical association of atherosclerosis and myocardial infarction risk

The relationship between atherosclerosis and the risk of myocardial infarction (MI) is well established, with a plethora of epidemiological data underscoring the impact of immune checkpoint inhibitors (ICIs) on cardiovascular events, particularly acute myocardial infarction. Recent studies have demonstrated a concerning trend in which patients undergoing treatment with ICIs exhibit an increased incidence of acute coronary events, including myocardial infarction, compared with those not receiving such therapies. For example, a retrospective analysis revealed that the use of ICIs is correlated with a heightened risk of atherosclerotic vascular events, including myocardial infarction and ischemic stroke, suggesting that these agents may exacerbate underlying atherosclerotic processes (73). This phenomenon is particularly relevant given the increasing use of ICIs in cancer therapy, necessitating a careful evaluation of cardiovascular risk factors in these patients. Furthermore, the inflammatory milieu associated with ICI therapy may contribute to the progression of atherosclerosis, thereby increasing the risk of acute coronary syndrome. The mechanisms underlying this relationship likely involve immune-mediated inflammation that disrupts vascular homeostasis, leading to plaque instability and subsequent thrombotic events. Additionally, the presence of traditional cardiovascular risk factors such as hypertension, hyperlipidemia, and diabetes in patients receiving ICIs may further compound the risk of MI, highlighting the need for vigilant cardiovascular monitoring in this population (74).

The exacerbation of inflammation and immune dysregulation in patients treated with ICIs is a critical area of research, as it may elucidate the pathways through which these therapies influence cardiovascular outcomes. For example, the inflammatory response triggered by ICIs can lead to an increase in circulating proinflammatory cytokines, which may play a role in destabilizing atherosclerotic plaques (75). Moreover, the interplay between the immune system and atherogenesis is complex, with recent studies indicating that immune cells, particularly T cells and macrophages, are integral to both the development of atherosclerosis and the inflammatory response following myocardial infarction. The activation of these immune pathways may not only enhance plaque formation but also contribute to the acute inflammatory response observed in MI, suggesting that targeting these pathways could be a potential therapeutic strategy to mitigate the cardiovascular risks associated with ICI therapy (76).

In conclusion, the clinical association between atherosclerosis and the risk of myocardial infarction is underscored by epidemiological data linking ICI therapy to increased cardiovascular events. The mechanisms of immune-mediated inflammation appear to play pivotal roles in this association, necessitating further research to develop strategies aimed at reducing cardiovascular risk in patients receiving ICI treatment. Comprehensive cardiovascular risk assessment and management should be integrated into the care of patients receiving ICIs to minimize the potential for adverse cardiovascular outcomes, including myocardial infarction and other ischemic events (77).

### 6.3 Early recognition and diagnostic strategies

Early recognition and diagnosis of myocardial infarction (MI) are critical for improving patient outcomes and minimizing complications. A comprehensive approach that integrates clinical manifestations, electrocardiographic (ECG) findings, and biomarker assessments is essential to accurately identify MI in its early stages. Clinically, patients may present with classic symptoms such as chest pain, dyspnea, and diaphoresis; however, atypical presentations, particularly in women and elderly individuals, can complicate the diagnosis (25). ECG remains a cornerstone of MI diagnosis, with ST-segment elevation indicating STEMI, whereas non-ST elevation MI (NSTEMI) may present with subtle changes that require careful interpretation. The use of serial ECGs can help identify evolving ischemic changes. Furthermore, cardiac biomarkers, particularly troponins, play pivotal roles in confirming MI, as they are highly sensitive and specific indicators of myocardial injury. Elevated levels of troponin I or T can be detected within hours of injury and remain elevated for several days, providing a critical diagnostic window for clinicians (28).

In addition to clinical and biochemical assessments, imaging techniques are increasingly recognized for their diagnostic value in MI. Echocardiography can provide real-time visualization of cardiac function, enabling the assessment of wall motion abnormalities that are indicative of ischemia or infarction. Advanced imaging modalities such as CMR can offer detailed insights into myocardial perfusion and viability, helping to differentiate between acute and chronic ischemic changes (29). Moreover, histological evaluations through endomyocardial biopsy may be warranted in cases where myocarditis is suspected, particularly in patients with recent immune checkpoint inhibitor therapy, which can mimic MI (78).

The integration of these diagnostic modalities allows for a more nuanced understanding of the underlying pathophysiology of MI, especially in the context of immune checkpoint inhibitors (ICIs). Recent studies have highlighted the role of the PD-1/PD-L1 pathway in cardiovascular diseases, suggesting that increased PD-L1 expression in the myocardium may serve as a biomarker for patients at risk of developing cardiac complications following ICI therapy (30). Therefore, a multidisciplinary approach that includes cardiology and oncology specialists is paramount for the early identification and management of cardiac complications, particularly in cancer patients receiving ICIs. This collaborative effort can increase the safety and efficacy of cancer treatments while addressing the cardiovascular risks associated with these therapies. In summary, the early recognition and diagnosis of myocardial infarction necessitate a comprehensive strategy that incorporates clinical assessment, ECG interpretation, biomarker analysis, and advanced imaging techniques, ultimately leading to improved patient outcomes and tailored therapeutic interventions.

### 6.4 Treatment principles for immune-related myocardial injury

The treatment of immune-related myocardial injury (IMI) necessitates a multifaceted approach that integrates immunosuppressive therapies, supportive care, and cardiac monitoring. Immunosuppressive treatments, particularly glucocorticoids and other immunosuppressive agents, have been shown to be effective in mitigating the inflammatory response associated with IMI. For example, high-dose corticosteroids, such as methylprednisolone, have rapidly reduced the levels of myocardial injury markers, including troponin I and N-terminal pro-brain natriuretic peptide (NT-proBNP), thereby improving clinical outcomes in patients experiencing immune checkpoint inhibitor-related myocarditis (79). The mechanism behind this efficacy involves the suppression of the overactive immune response that characterizes IMI, which can lead to significant myocardial damage if left unchecked. In addition to glucocorticoids, other immunosuppressive agents may be utilized to further control inflammation, particularly in patients who are resistant to steroid therapy. For example, the combination of immune checkpoint inhibitors with agents such as tocilizumab has shown promise in reducing inflammatory myocardial injury by targeting specific cytokine pathways (80). Furthermore, supportive therapies, including fluid management, electrolyte balance, and the use of inotropic agents, are essential for maintaining cardiac function during acute episodes of IMI. Continuous cardiac monitoring is critical to detect any deterioration in cardiac function promptly, allowing timely interventions that can prevent adverse outcomes. The integration of these treatment modalities aims to balance the need for immunosuppression with the preservation of cardiac function, thus optimizing patient outcomes in the setting of immune-related myocardial injury.

Supportive care plays an equally vital role in the management of IMI, particularly in the context of heart failure and arrhythmias that may arise as complications of myocardial injury. Patients with IMI often present with varying degrees of cardiac dysfunction, necessitating close monitoring and management of heart failure symptoms. This includes the use of diuretics to manage fluid overload and the careful titration of heart failure medications, such as angiotensin-converting enzyme (ACE) inhibitors or beta-blockers, to optimize cardiac output and reduce myocardial workload (81). Additionally, the implementation of advanced cardiac monitoring techniques, such as echocardiography and CMR, can provide valuable insights into cardiac structure and function, guiding therapeutic decisions (82). It is also crucial to address potential complications, such as arrhythmias, which can significantly impact mortality in patients with IMI. The use of antiarrhythmic medications or temporary pacing may be warranted in cases of significant arrhythmias, ensuring that patients are stabilized and monitored closely for any changes in their clinical status.

In summary, the treatment of immune-related myocardial injury requires a comprehensive strategy that encompasses immunosuppressive therapies, supportive care, and vigilant cardiac monitoring. ICI-related myocarditis frequently leads to MACE, which is associated with poor prognosis, however, by effectively managing the inflammatory response and providing supportive interventions, clinicians can improve outcomes for patients suffering from this complex condition. Ongoing research into the mechanisms of IMI and the development of targeted therapies will further enhance our ability to treat this challenging complication of immunotherapy and other immune-mediated conditions.

### 6.5 Multidisciplinary collaborative model

The collaboration between cardiology and oncology is becoming increasingly vital in managing patients with complex conditions such as myocardial infarction (MI) and cancer. The intersection of these two fields is particularly relevant given the increasing use of immune checkpoint inhibitors (ICIs), such as PD-1/PD-L1 inhibitors, which are associated with cardiovascular complications. A multidisciplinary approach allows for a comprehensive evaluation of the patient's overall health status, including both cardiac and oncological aspects, ensuring that treatment strategies are tailored to the individual needs of the patient. This collaboration facilitates the identification of potential cardiovascular risks associated with cancer therapies, enabling proactive management strategies that can mitigate adverse events. For example, patients receiving ICIs may experience immune-related adverse events, including myocarditis or myocardial infarction, which necessitate close monitoring and timely intervention by both cardiologists and oncologists (28, 29). By working together, these specialists can develop integrated care pathways that address the unique challenges faced by patients with concurrent cardiovascular and oncological conditions, ultimately improving patient outcomes and quality of life.

In designing individualized treatment plans, it is crucial to conduct thorough risk assessments that consider the patient's cancer type, stage, treatment history, and existing cardiovascular conditions. This risk stratification can guide the selection of appropriate therapeutic interventions, including the choice of ICIs and the potential need for cardioprotective measures. For example, certain patients may benefit from the addition of cardioprotective agents or the implementation of lifestyle modifications to reduce the risk of cardiovascular events during cancer treatment (83, 84).

Furthermore, ongoing communication between cardiologists and oncologists is essential for monitoring treatment responses and adjusting therapeutic strategies as needed. This collaborative framework not only enhances the safety and efficacy of cancer treatments but also fosters a holistic approach to patient care that prioritizes both oncological and cardiovascular health. We therefore advocate for the inclusion of highly specialized cardio-oncology experts within these multidisciplinary teams to ensure precise monitoring and management. The integration of such teams in clinical practice is thus a promising strategy for addressing the complexities of managing patients with concurrent cardiac and oncological conditions, paving the way for improved therapeutic outcomes and patient satisfaction.

## 7 Future research directions and clinical translation prospects of immunotherapy for myocardial infarction

### 7.1 Exploration and validation of novel immune checkpoint targets

The investigation of alternative immune checkpoint targets, such as lymphocyte activation gene 3 (LAG-3) and CD47, has gained momentum as a potential strategy for cardiovascular protection, particularly after myocardial infarction (MI). LAG-3 is a checkpoint molecule that regulates T-cell activity and contributes to the balance between immune activation and tolerance in cancer, autoimmunity, and other diseases. Evidence indicates that inhibiting LAG-3 can enhance T-cell–mediated repair, reduce inflammation, and improve cardiac function following MI (85). Similarly, CD47—known as the “don't eat me” signal—prevents macrophage phagocytosis. In MI, CD47 blockade facilitates the clearance of apoptotic cells and promotes reparative macrophage activity, thereby limiting myocardial damage and supporting functional recovery (86). Targeting these pathways simultaneously may offer a synergistic benefit by restoring immune balance and promoting more effective cardiac healing.

Nonetheless, combining novel checkpoint inhibitors requires careful evaluation of both therapeutic potential and associated risks. Heightened immune activation can lead to adverse effects, particularly in patients with underlying cardiovascular disease. For instance, while blocking LAG-3 and CD47 may boost immune responses and support tissue repair, it also carries the possibility of excessive inflammation and collateral damage in the myocardium or other organs (82). Therefore, clinical application demands a careful balance between efficacy and safety. Current preclinical and clinical studies will be critical to define the therapeutic window, assess toxicity profiles, and validate cardiovascular benefits. The ultimate objective is to design immunotherapeutic strategies that optimize cardiac protection and repair while minimizing immune-related complications, offering safer and more effective options for patients with MI and other cardiovascular disorders.

### 7.2 Immune regulation and innovative therapeutic strategies for myocardial repair

The intersection of immune regulation and myocardial repair represents a promising frontier in the treatment of myocardial infarction (MI). Recent advancements in cellular therapies, gene editing, and the use of immune modulators have opened new avenues for enhancing cardiac repair processes. Cellular therapies, particularly those utilizing mesenchymal stem cells (MSCs), have shown potential in modulating inflammatory responses and promoting angiogenesis in the postinfarction heart. MSCs secrete a variety of trophic factors that can improve cardiac function and facilitate tissue regeneration through paracrine signaling mechanisms (87). Furthermore, the integration of gene editing technologies, such as CRISPR/Cas9, allows for precise modifications in cardiac cells, potentially enhancing their regenerative capabilities and immune tolerance. For example, gene editing can be employed to knock down proinflammatory cytokines or enhance the expression of protective factors, thereby creating a more favorable environment for cardiac repair (88). The combination of these innovative approaches, including cellular therapies and gene editing, may yield synergistic effects, ultimately leading to improved outcomes in patients recovering from MI.

In parallel, the development of novel pharmacological agents that target inflammation and immune tolerance is crucial for advancing therapeutic strategies for myocardial repair. Recent studies have highlighted the role of immune checkpoint inhibitors, such as PD-1/PD-L1, in modulating immune responses during myocardial injury (11). These pathways can be harnessed to promote immune tolerance, thereby reducing adverse inflammatory responses that can exacerbate cardiac damage after MI. For example, agents that selectively inhibit proinflammatory pathways while enhancing anti-inflammatory responses may mitigate the detrimental effects of excessive immune activation during the healing process. Additionally, the exploration of small molecules and biologics that target specific inflammatory mediators, such as IL-1β and TNF-α, has shown promise in preclinical models (89). These agents can be combined with existing therapies to create a multifaceted approach that not only addresses the immediate needs of myocardial repair but also fosters long-term cardiac health.

Moreover, the identification of biomarkers that predict immune responses and repair outcomes post-MI is essential for tailoring these innovative strategies to individual patients. Biomarkers can guide clinicians in selecting appropriate therapies and monitoring their effectiveness, thereby optimizing treatment regimens. For example, elevated levels of specific cytokines or immune cell populations may indicate an ongoing inflammatory process that requires intervention (5). The integration of biomarker-driven approaches with advanced therapeutic modalities, including cellular therapies and immune modulators, can increase the precision of treatment strategies aimed at myocardial repair.

In summary, integrating immune modulation with emerging therapeutic innovations offers significant promise for enhancing recovery in patients with myocardial infarction. Approaches that combine cell-based therapy, genetic engineering, and precision pharmacological agents may provide a multifaceted framework for cardiac repair, addressing the intricate balance between inflammatory injury and tissue regeneration. As the field progresses, tailoring interventions to individual immune profiles and reparative pathways is likely to become a cornerstone of cardiovascular therapy. Rigorous clinical investigations will be required to confirm the safety and effectiveness of these strategies and to determine their capacity to improve myocardial healing and overall cardiac performance.

### 7.3 Clinical trial design and biomarker development

The design of clinical trials focusing on the PD-1/PD-L1 immune checkpoint pathway in the context of myocardial infarction (MI) necessitates a robust framework for identifying and monitoring high-risk patients. Patients with preexisting cardiovascular conditions, such as coronary artery disease, heart failure, or a history of myocardial infarction, represent a particularly vulnerable population when exposed to immune checkpoint inhibitors (ICIs) (90). The stratification of these patients on the basis of their medical history, demographic factors, and specific tumor characteristics is essential for tailoring treatment approaches and mitigating the risk of immune-related adverse events (irAEs). For example, studies have indicated that younger patients and those with a higher body mass index may be at increased risk for irAEs, particularly when treated with PD-1/PD-L1 inhibitors (90). Furthermore, the incorporation of comprehensive screening tools and biomarkers into clinical trial designs can enhance the identification of at-risk individuals. Biomarkers such as circulating blood counts, cytokines, and autoantibodies can provide valuable insights into a patient's immune status and potential susceptibility to adverse events, thereby guiding treatment decisions and monitoring strategies (90).

In addition to patient selection, the establishment of an early warning system for immune-related myocardial injury is crucial in the context of clinical trials involving PD-1/PD-L1 inhibitors. Early detection of cardiotoxicity can significantly improve patient outcomes by allowing timely intervention and the management of complications. Current diagnostic modalities, including electrocardiograms, cardiac biomarkers, and advanced imaging techniques such as CMR, play a pivotal role in this early warning framework (91). However, the challenge lies in the nonspecific nature of symptoms associated with cardiotoxicity, which can complicate timely diagnosis. Therefore, an integrated approach that combines clinical assessment with biomarker profiling may increase the sensitivity and specificity of early detection systems. For example, elevated levels of cardiac troponins or changes in the neutrophil‒lymphocyte ratio have been associated with an increased risk of myocarditis and other cardiovascular complications in patients receiving ICIs (90, 91).

Moreover, the development of a comprehensive monitoring protocol that includes both clinical and laboratory parameters is essential for the effective management of patients undergoing treatment with PD-1/PD-L1 inhibitors. This protocol should encompass regular assessments of cardiac function and biomarkers, allowing for the identification of any deterioration in cardiac status. Collaborative efforts among oncologists, cardiologists, and primary care providers are vital to ensure that patients are monitored closely throughout their treatment journey, particularly in the peri-operative setting where the risk of adverse events may be heightened (91). As the landscape of immunotherapy continues to evolve, ongoing research into the mechanisms underlying cardiotoxicity, as well as the identification of novel biomarkers, will be critical in refining clinical trial designs and enhancing patient safety. Ultimately, the integration of these strategies into clinical practice will not only improve the management of high-risk patients but also contribute to the overall success of immunotherapeutic interventions in the treatment of malignancies associated with cardiovascular comorbidities (92).

## 8 Conclusion

In conclusion, the PD-1/PD-L1 immune checkpoint pathway is a critical player in the pathophysiology of myocardial infarction, influencing both myocardial inflammatory responses and subsequent repair processes. Continued exploration of this pathway will be essential in refining immunotherapy approaches, ultimately leading to improved patient care and outcomes in the management of myocardial infarction and related cardiovascular events. As we move forward, the commitment to personalized medicine and the integration of interdisciplinary insights will be paramount in navigating the complexities of immune modulation in cardiovascular disease.
